# On the Evolution of Temperature and Combined Stress in a Work Roll under Cyclic Thermo-Mechanical Loadings during Hot Strip Rolling and Idling

**DOI:** 10.3390/ma13215054

**Published:** 2020-11-09

**Authors:** Kejun Hu, Qinghe Shi, Wenqin Han, Fuxian Zhu, Jufang Chen

**Affiliations:** School of Materials and Engineering, Jiangsu University of Technology, Changzhou 213001, China; qhshi@jsut.edu.cn (Q.S.); hwq@jsut.edu.cn (W.H.); jxzfx@jsut.edu.cn (F.Z.); jfchen@jsut.edu.cn (J.C.)

**Keywords:** hot rolling, work roll, thermo-mechanical stress, residual stress redistribution, FEM

## Abstract

An accurate prediction of temperature and stress evolution in work rolls is crucial to assess the service life of the work roll. In this paper, a finite element method (FEM) model with a deformable work roll and a meshed, rigid body considering complex thermal boundary conditions over the roll surface is proposed to assess the temperature and the thermal stress in work rolls during hot rolling and subsequent idling. After that, work rolls affected by the combined action of temperature gradient and rolling pressure are investigated by taking account of the hot strip. The accuracy of the proposed model is verified through comparison with the calculation results obtained from the mathematical model. The results show that thermal stress is dominant in the bite region of work rolls during hot rolling. Afterwards, the heat treatment residual stresses which are related to thermal fatigue are simulated and introduced into the work roll as the initial stress to evaluate the redistribution under the thermal cyclic loads during the hot rolling process. Results show that the residual stress significantly changed near the roll surface.

## 1. Introduction

Work rolls made of high-speed steel (HSS) are widely used in finishing stands of hot strip rolling mills because of their excellent hardness, good wear resistance and high temperature properties [1,2]. During hot rolling, work rolls are alternately exposed to mechanical and thermal loads due to contact with a hot strip and cooling water in every revolution. Consequently, the cyclic thermal stress caused by severe temperature gradients and the mechanical stress caused by rolling pressure and the contact action with the back-up rolls, are imposed on the roll surface. Once the magnitude of the thermal and mechanical stresses exceed the hot yield strength of the roll material, the roll surface is likely to crack due to thermal fatigue combined with tribological factors such as abrasion, adhesion and oxidation. If these cracks are not removed by an appropriate roll dressing program, they can lead to the propagation of larger cracks, and subsequent spalling of roll surface fragments may occur [3,4]. Thermal fatigue and its associated roll surface failure mechanisms have direct negative effects on the service life of the work rolls and the surface quality of the finished products. Thus, accurate understanding of the temperature and stresses in work rolls is crucial to predict service life of the work rolls.

The thermal behavior of the work rolls in a rolling mill has always been of great interest to researchers [5]. Thus, several works have been conducted to predict temperature and stresses in the work rolls, in which various methods and techniques were utilized such as the analytical approach, the finite-difference method (FDM) and the finite element method (FEM). Steven et al. [6] firstly measured the temperature distribution in work rolls during industrial hot rolling using thermocouples and investigated the effect of cooling practice on roll life. A mathematical model used for determining steady-state temperature distribution in a rotating roll subjected to constant surface heat flux over one portion and convective cooling over another portion was presented by Patula [7]. Tseng et al. [8,9] developed an uncoupled analytic thermomechanical solution to determine the temperature field and the thermal stresses in rolls. Lai et al. [10] investigated the transient thermal stress of work rolls during strip rolling using a coupled thermo-elasticity analytical method. Yiannopoulos et al. [11] introduced an internal pressure to the inside face of a hollow cylinder to investigate temperature and thermal stress distributions in the roll. Three- and two-dimensional heat transfer in the work rolls of a hot strip rolling stand were considered by Lin and Chen [12]. Shangwu et al. [13] employed a combined finite element-boundary element approach to achieve a stress field within work rolls during plane strain rolling. Chang [14] employed the finite-difference method coupled with the assumptions of a steady-state rolling condition and a nonuniform heat flux in the deformation zone to predict the work roll temperature profile and the resulting thermal stresses. The transient thermal behaviors of work rolls were also conducted using a finite element method. For example, Guo and Hwang et al. [15,16] presented a two-dimensional (2D) transient model for calculating the temperature distribution in work rolls. Lee et al. [17] employed a three-dimensional (3D) finite element under unsteady-state rolling conditions to predict the thermal history within the work rolls during deformation as well as during the idling cycles. Hsu et al. [18] proposed an FEM model to predict the surface thermal behavior of work rolls during hot rolling. Sun et al. [19] and Li et al. [20,21] presented a thermo-mechanical model to consider the thermo-mechanical response of work rolls in a hot strip rolling process. Recently, Serajzadeh et al. [22,23,24] developed a two-dimensional FEM model with work rolls and hot strips to predict the temperature and the thermo-mechanical stress in work rolls during the hot slab rolling process. Fischer et al. [25] proposed a solution with a moving heat source to investigate the temperature and the stress near the roll surface. Benasciutti et al. [26,27,28] completed a series of studies to estimate the transient temperature, thermal stress and the fatigue life of a work roll using a two-dimensional FEM model even a one-dimensional harmonic FEM model. Thermal and mechanical stresses produced in the work rolls during the hot rolling process are predicted using a thermoplastic finite element approach in the ABAQUS standard software by Qayym et al. [29], comparing with a cold rolling case. An integrated mathematical model was developed to study the thermo-mechanical behavior of strips and work rolls during the warm rolling process of steels by Koohbor [30]. With regard to HSS rolls, Gao et al. [31] studied temperature variations and compared them with the results from a high-Cr roll using the FEM model. Dünckelmeyer et al. [32] proposed an analytical model to predict the surface thermo-mechanical residual stresses in work rolls and the plastic strains were described to discuss the influences of rolling speed and strip temperature. Na et al. [33] analyzed the temperature evolutions and resulting stress changes of work rolls during both rolling and idling at the last stand of a finishing mill in the actual hot strip rolling process to examine the critical idling time for the work rolls between strips. Deng et al. [34,35] investigated temperature and thermal stress to evaluate the oxidation behavior of high-speed steel work rolls.

Regarding publications dealing with the modeling of strip rolling processes, in spite of several of these concentrating on temperature and stresses, the combined impacts, including thermal stress, mechanical stress and heat treatment residual stress, need further study for a better understanding of the service life of work rolls in practical industrial service condition during the hot rolling process. In addition, most research on stress in the rolling process is devoted to the thermo-elastic behavior of work rolls that are only investigated with very few revolutions. There are few numerical works on thermo-plastic assessment of HSS rolls, especially for a practical hot rolling process including rolling and idling. The present study attempts to investigate temperature and thermal stress evolution, thermo-mechanical stress and redistribution of heat treatment residual stress in practical industrial service conditions. Hence, a 2D model based on the near contact method with good accuracy and with a relatively low computational cost is proposed, which is applicable for simulating the thermo-mechanical transient with a large number of roll revolutions. Firstly, the transient thermal analysis using temperature dependent thermal properties for both the work roll and hot strip, is carried out to evaluate temperature and thermal stress in work roll during rolling and idling. Then, the simulation of thermo-mechanical stress caused by the combined action of the temperature gradient and rolling pressure is performed to compare with the thermo-stress. In order to verify FEM model, a comparison is made between the roll force predicted by the FEM model and the calculation results obtained from mathematical model. In order to investigate the thermal residual stress during hot rolling, a long idling time about 100 s is conducted to completely cool work roll. Finally, the heat treatment residual stresses that are related to thermal fatigue, are simulated and introduced into work roll as the initial stress in order to investigate the redistribution of under the cyclic load during hot rolling.

## 2. FEM Simulations

Generally, the 3D model of work rolls with hot strip can provide a very realistic representation of the rolling process, such as temperature, stress distributions and deformation along the axis, but this model is at high computational costs. For thermal fatigue damage assessment, it is most important to simulate the work roll response over a long rolling period, so that the calculated stress and strain are fully representative of the stabilized cyclic material behavior. In fact, thermal fatigue damage is mainly caused by the circumferential and radial stresses and strains at work roll surface, whereas it is little affected by the stress–strain along the roll axis. Therefore, although some simplification along the axis, a 2D model work roll can well predict the work roll fatigue life because this model shows the capability to predict—with sufficient precision distribution—the temperature distribution, the work roll thermal expansion and the thermo-mechanical stresses and strains. Furthermore, considering a strong reduction in the computational cost, the 2D model is more applicable for simulating the thermo-mechanical transient with a large number of roll revolutions to have an accurate mapping of the stress–strain time evolution that is absolutely crucial to perform a thermal fatigue life assessment.

### 2.1. FEM Model Description

**Figure 1:**
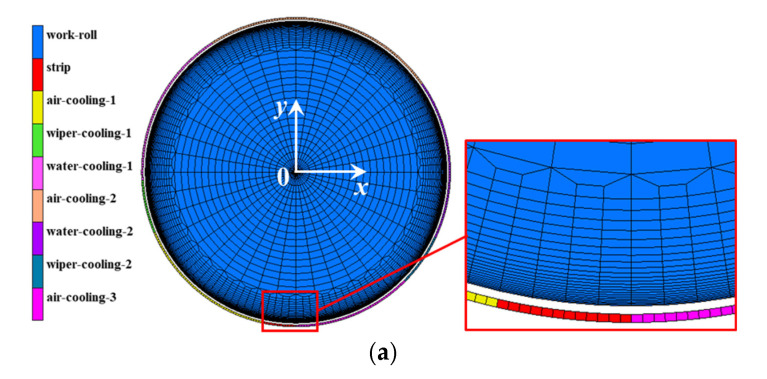

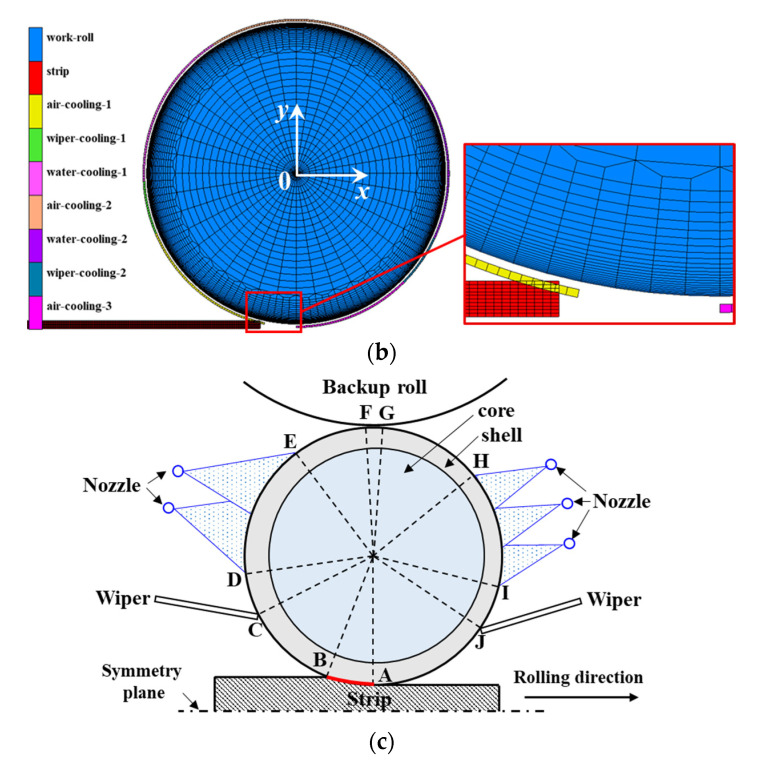
(**a**) FEM model without hot strip used for thermal stress analysis, (**b**) FEM model with hot strip used for thermo-mechanical stress analysis, (**c**) schematic of thermal and mechanical boundary conditions of the work roll during hot strip rolling.

A 2D rolling model without a hot strip (see Figure 1a) and a model with a hot strip (see Figure 1b) have been developed to simulate the thermal stress and the thermo-mechanical stress during the hot strip rolling process respectively, using the commercial finite element software MSC.Marc/MENTANT. In both the strip and work roll, a four-node bilinear plane strain element was used for the thermo-mechanical coupling analysis, considering von Mises plasticity with criterion and the isotropic strain hardening rule. The nonlinear thermo-mechanical coupled analysis was performed by means of an implicit solver with a modified full Newton–Raphson method. A mesh refinement was imposed near the surface along both the circumferential and radial directions to accurately describe the large temperature and stress gradient in solution variables during hot rolling. The model has an axisymmetric mapped mesh, with a total of 7888 elements and 8590 nodes.

Figure 1c illustrates the thermal boundary conditions along the circumferential direction of the work roll during one revolution. Regions A~B involve high heat flow between the work roll and hot strip; regions B~C, E~H and J~A involve natural air cooling without the strip radiation at the entrance and exit, as well as the heat exchange between the work roll and backup roll; regions C~D and I~J involve wiper cooling; regions D~E and H~I involve water spraying cooling. Recently, the heat transfer analysis between a work roll and surrounding objects was generally conducted by using the APDL script language or the film/flux subroutines, which were often confidential [20,21,26,27,34,35]. In order to solve this problem, in this paper, a heat transfer model was performed based on the near contact method using a meshed, rigid body near the roll surface to predict roll temperature and the thermal stress evolution during hot strip rolling. In the simulation of thermal stress analysis without a hot strip, shown in Figure 1a, the heat transfer type meshed, rigid body layer with a larger radius was established surrounding the deformable work roll, and then the different temperatures were defined for the meshed, rigid body in different regions. The heat transfer coefficients depending on the different regions were defined as a function of the distance between the work roll surface and the meshed rigid body. The thermal boundary conditions along the circumferential direction of the work roll during rolling can be achieved by setting a specified rotating speed for the meshed, rigid body layer. In the simulation of thermo-mechanical stress analysis with hot strip shown in Figure 1b, the elements of the meshed, rigid body in regions A~B were removed and the meshed hot strip was established to realize the heat transfer and mechanical action between the work roll and hot strip.

### 2.2. Heat Transfer Description

During the front finishing stands in a hot strip mill, the temperature near the surface of the work roll rises rapidly due to the heat transfer through the steel plate and drops quickly due to water sprays and air cooling. The general heat transfer equation in cylindrical coordinates for the unsteady state of a work roll can be given as [22,23,36]:

(1) $$\rho_{r}C_{r}\frac{\partial T}{\partial t}=\frac{1}{r}\frac{\partial }{\partial r}(k_{r}r\frac{\partial T}{\partial r})+\frac{1}{r^{2}}\frac{\partial }{\partial \theta }(k_{r}\frac{\partial T}{\partial \theta })+\frac{\partial }{\partial z}(k_{r}\frac{\partial T}{\partial z})+\overset{•}{Q}$$

where $\rho_{r}$, $C_{r}$ and $k_{r}$ are the density, the specific heat and the thermal conductivity of work roll, respectively; r, θ and z are the radial, the circumferential and the longitudinal coordinates; T is temperature; *t* is the time; and $\overset{•}{Q}$ is the rate of heat generation caused by the deformation, which is negligible in this study due to its small effect on roll temperature. In addition, it is assumed that heat conduction in the longitudinal direction of the work roll can be neglected. Thus, the two-dimensional unsteady heat conduction equation in the cylindrical coordinate can be simplified as follows:

(2) $$\rho_{r}C_{r}\frac{\partial T}{\partial t}=\frac{1}{r}\frac{\partial }{\partial r}(k_{r}r\frac{\partial T}{\partial r})+\frac{1}{r^{2}}\frac{\partial }{\partial \theta }(k_{r}\frac{\partial T}{\partial \theta })$$

The initial thermal boundary condition of the work roll can be expressed as:

(3) $$T(r,\;\theta ,\;0)=T_{0}$$

where $T_{0}$ is the initial temperature of work roll and *T*_0_ = 30 °C before the hot rolling in this study.

Since only the upper half of the hot strip is modeled, the thermal insulation is applied in the symmetry plane of the hot strip in the thermo-mechanical stress analysis as

(4) $$-k_{s}\frac{\partial T}{\partial y}=0$$

According to Newton’s cooling law [24], the amount of heat exchanged between a body and its environment depends on the difference between the roll surface temperature and the environment temperature. The relevant heat flux density including the heat loss due to radiation and convection can be given by

(5) $$-k_{r}\frac{\partial T}{\partial r}=h^{*}(T-T_{s})\;+q_{r\;}$$

where $h^{*}$ is the convection heat transfer coefficient that depends on the positions of the work roll surface; *T*_s_ is the temperature of the work roll surface; and $q_{r\;}$ means the heat lost due to radiation and is negligible in this study.

In the bite regions (A~B), the heat transfer coefficient $h_{\mathrm{con}}$ was calculated using an empirical equation considering the rolling pressure and the surface flow stress of the work piece derived by Hlady et al. [37]:

(6) $$hcon=\frac{k}{C}\frac{P_{r}}{\sigma (T_{s},\bar{\dot{\varepsilon }})}$$

(7) $$k=\frac{k_{r}k_{s}}{k_{r}+k_{s}}$$

where *C* = 35 × 10^−6^ m and is a general roughness term; *k*_s_ is the thermal conduction of the strip; *P*_r_ is the rolling pressure; and *σ* is the flow stress of strip calculated using the equation derived in Refer [38,39].

In the air cooling regions (B~C, E~H and J~A) the heat transfer coefficients *h*_air_ were calculated using the following equation [40,41]:

(8) $$h_{air}=1.456{\left(T_{s}-T_{a}\right)}^{1/3}$$

where *T*_a_ is the air temperature.

In the wiper cooling regions (C~D and I~J) similar calculation equations of heat transfer coefficients were proposed in [34,35,41]:

(9) $$h_{\mathrm{wiper}}=0.023R_{e}^{0.8}P_{\mathrm{rcw}}^{0.4}\frac{k_{\mathrm{cw}}}{l_{c}}$$

(10) $$R_{e}=\nu_{w}l_{c}/\upsilon_{\mathrm{cw}}$$

(11) $$P_{\mathrm{rcw}}=\rho_{\mathrm{cw}}c_{\mathrm{cw}}\upsilon_{\mathrm{cw}}/k_{\mathrm{cw}}$$

where *R*_e_ is the Reynolds number; *P*_rcw_ is the water Prandtl number; *k*_cw_ is the water thermal conductivity; *l*_c_ is the contact arc length along the roll surface of the wiper region; *v*_r_ is the roll velocity; $\upsilon_{\mathrm{cw}}$ is the water kinematic viscosity; $\rho_{\mathrm{cw}}$ is the water density; and $c_{\mathrm{cw}}$ is the water specific heat.

In the spray cooling regions (D~E and H~I) the heat transfer coefficients considering the roll temperature can be given by [34,35,41]

(12) $$h_{\mathrm{cw}}=6870\times Q^{0.19}P_{\mathrm{sp}}^{0.27}\;(T_{r}\leq 100{}^{\circ}C)$$

(13) $$h_{\mathrm{cw}}=2.9\times 10^{6}\times Q^{0.08}P_{\mathrm{sp}}^{0.05}\frac{B}{200-T_{\mathrm{wa}}}\times \frac{4815.5}{3600}\;(T_{r}\geq 100{}^{\circ}C)$$

(14) $$B\;=\;{(T_{\mathrm{wa}}/16)}^{-0.17}\;(Q\geq 10000\;(l\cdot s^{-1}\cdot m^{-2}))$$

(15) $$B\;=\;1.0\;(Q\leq 10000\;(l\cdot s^{-1}\cdot m^{-2}))$$

where *Q* = *V*_sp_/*A*_sp_ is the water flow per unit area; *V*_sp_ is the water flow; *A*_sp_ is the water flow area; *P*_sp_ is the water pressure; and *T*_wa_ is the cooling water temperature.

The heat transfer coefficients used in this study were calculated based on the practical industrial hot rolling parameters given in Table 1.

### 2.3. Material Properties Description

Material properties of the work roll consist of high-speed steel (HSS) and ductile cast iron (DCI). Those used in this study were measured experimentally at room temperature and 100–600 °C with an interval of 100 °C, including Young’s modulus, Poisson’s ratio, thermal expansion coefficient, conductivity, density and specific heat, all as a function of the temperature. The plastic behavior of material is defined by stress–strain curves from compressive and tensile tests as a function of temperature. The experimentally measured material properties at room temperature are listed in Table 2.

In this study, a hot strip of Q235 class steel with an initial thickness of 44 mm and 964 mm width was rolled in the finishing stand mills. The elastic isotropic material is defined using the temperature-dependent elastic modulus and Poisson’s ratio. The flow stress influenced by temperature, strain and strain rate, and is crucial to define the thermo-plastic behavior of the hot strip during hot rolling. In this paper, the regression model of deformation resistance for the hot strip is determined by the equation as [38]:

(16) $$\sigma =150\exp(-2.8685T+3.6573){\left(\frac{\dot{\varepsilon }}{10}\right)}^{0.2121T-0.1531}\left[1.4403{\left(\frac{\varepsilon }{0.4}\right)}^{0.3912}-(1.4403-1)\frac{\varepsilon }{0.4}\right]$$

where *T* = (*t* + 273)/1000; *t* is the deformation temperature; $\dot{\varepsilon }$ is the deformation rate; and ε is the deformation degree. Equation (16) can be entered into the finite element software MSC.Marc/MENTANT as a function to generate the data points as a table. Here, the created table with three independent variables (temperature, equivalent plastic strain and equivalent plastic strain rate) is applied to define the material properties of the hot strip.

## 3. Results and Discussion

### 3.1. Temperature and Thermal Stress of Work Rolls during Rolling and Idling

**Figure 2:**
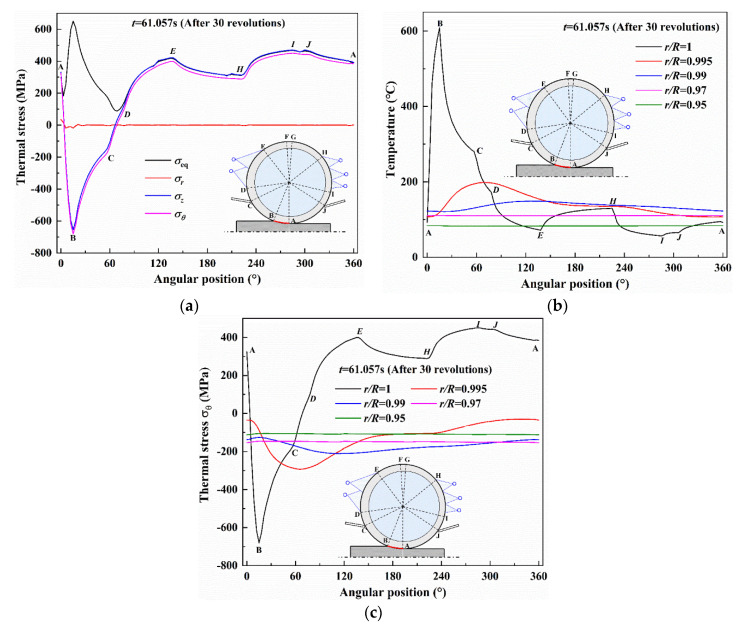
Distribution of temperatures and thermal stresses as a function of the angular position after 30 revolutions: (**a**) temperatures with the different depths, (**b**) thermal stress components at the roll surface; (**c**) thermal stresses with the different depths.

Figure 2 shows the distributions of the temperature and the thermal stress σ_θ_ with different depths versus angular coordinate *θ* after 30 revolutions. *R* means the roll radius and *r* means the distance from the roll center. As shown in Figure 2a, as expected, surface temperature varies severely due to contact with the hot strip and water cooling. It is interesting to note that the maximum temperature and the temperature change range near the surface decrease significantly with the increase of depth, particularly in r/R = 0.97 and 0.95. It can be found that the surface temperature increases rapidly in the bite region and the maximum temperature occurs in the exit *B* of the bite region (regions A~B). As the work roll releases from the bite region, the surface temperature decreases continuously as it comes into contact with air cooling (regions B~C), wiper cooling (regions C~D) and spray water cooling (regions D~E), respectively. The surface temperature, by contrast, increases abruptly in the subsequent air cooling regions (regions E, H and J~A).This phenomenon can be attributed to the lower convection heat transfer from air compared with the higher conductive heat transfer from the subsurface layer which has the higher temperature. Figure 2b illustrates the surface thermal stress components in radial, circumferential and axial directions and the equivalent von Mises stress. A typical multiaxial state of the thermal stress can be observed. Radial stress *σ_r_* is equal to zero, even though a very small value fluctuation occurs in the bite region. Instead, circumferential stress *σ_θ_* and axial stress *σ_z_* vary significantly with the angular position, and they are almost identical in value.

The induced thermal stress corresponding to the temperature distribution is further observed in Figure 2c. The thermal stress with different depths shows a similar tendency of the temperature, namely that the significant range of thermal stress develops in the roll surface but the range is much less in the subsurface. During the bite region (regions A~B), the roll surface produces expansion deformation with a temperature increase but it would become constrained by the surrounding material with a lower temperature, resulting in high compressive thermal stress. The peak value of thermal stress is produced in the exit B where maximum temperature occurs. In the subsequent cooling regions (regions B~E, and H~I), the compressive stress continues to decrease even turning to a tensile state due to the contraction deformation at the surface with temperature decreases. It is worth noting that the tensile thermal stress occurs in the wiper region (regions C~D), while the produced tensile stress is not typical in previous studies which focused on the thermo-elastic behavior of work rolls with a relatively low initial roll temperature of 30 °C [33,34,35]. The compressive stress during the bite region can even reach the yielding stress of the roll material in high temperature, leading to plastic deformation and subsequent tensile stress during the cooling region. The plastic deformation and the induced tensile stress are associated with roll fatigue life, generally, causing roll failure from a low cycle thermal fatigue. It should be noted that the cyclic plastic deformation does not happen in the unloading process in the cooling region. This could be contributed to the yielding stress of the roll material, which obviously continuously increases with temperature decreases. In the subsequent air cooling regions (regions E~H and J~A), the tensile stress slightly decreases due to the increase of surface temperature in these two regions. It can be seen that maximum tensile stress occurs at point *I* where surface temperature is lowest.

**Figure 3:**
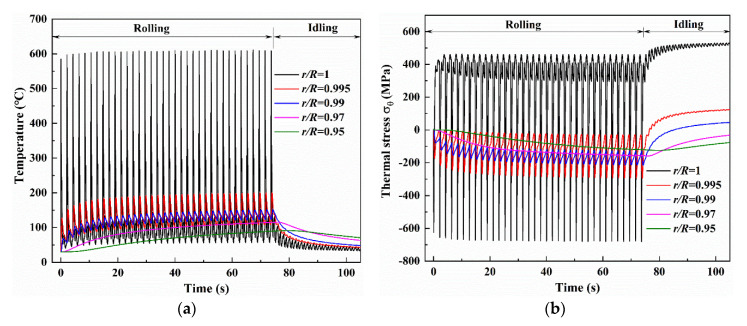
Histories of temperature and thermal stress with the different depths during the whole hot rolling of one strip and the subsequent idling: (**a**) temperatures, (**b**) thermal stresses.

More complete information of temperature and thermal stress is given in Figure 3, which displays the histories of temperature and thermal stress with the different depths during the whole hot rolling process of one strip and the subsequent idling. As shown in Figure 3a, during the rolling process the maximum surface temperature gradually increases in several early revolutions, and then reaches a steady temperature range with a maximum value of 605 °C and a minimum value of 56 °C. By contrast, the continuous increases of the maximum temperature and the minimum temperature are observed at the depth of *r*/*R* = 0.995 and 0.99, with smaller amplitudes of temperature variation or even approximate smooth curves at the depths of r/R = 0.97 and 0.95. After one strip rolling, the maximum temperatures reach 200 °C, 151 °C, 117 °C and 92 °C at the r/R = 0.995, 0.99, 0.97 and 0.95, respectively. It should be noted that the cooling conditions in the spray water cooling, wiper cooling and air cooling during the subsequent idling are just the same as those in rolling, but the bite region is replaced with air cooling. The temperatures at r/R = 1, 0.995, 0.99 and 0.97 decrease gradually, and the decreasing rates are slower with increased distance from the surface. Different to the temperatures at r/R = 1, 0.995, 0.99 and 0.97, the temperature slightly increases and then decreases at r/R = 0.95. This phenomenon can be attributed to the heat transfer from being closer to the surface than to the roll inner. At the end of the idling (about 30 s), the temperatures reach 33 °C, 42 °C, 48 °C, 63 °C and 71 °C at the r/R = 1, 0.995, 0.99, 0.97 and 0.95, respectively. This result indicates that the idling time (about 30 s) given in finishing stand F1 is enough to reduce surface temperature but that the subsurface temperature is still high.

Compared with the temperatures in the work roll during rolling, as shown in Figure 3a, a similar variation trend is observed in thermal stress $\sigma_{\theta }$ which shows a great temperature amplitude in the roll surface of r/R = 1 but very small temperature amplitudes in the roll subsurface of r/R = 0.995, 0.99, 0.97 and 0.95. After several early revolutions, a stable maximum compressive stress of −681 MPa and a stable maximum tensile stress of 460 MPa that are achieved at the roll surface correspond to the stable temperature state. The thermal stresses in the roll subsurface of r/R = 0.995, 0.99, 0.97 and 0.95, by contrast, remain in compression at all times during rolling. During the following idling, tensile stresses gradually increase depending on whether the roll surface or subsurface due to thermal shrinkage is accompanied by temperature decrease. At the end of the idling, the thermal stresses reach 530 MPa, 125 MPa, 46 MPa, −33 MPa and −77 MPa at the r/R = 1, 0.995, 0.99, 0.97 and 0.95, respectively.

**Figure 4:**
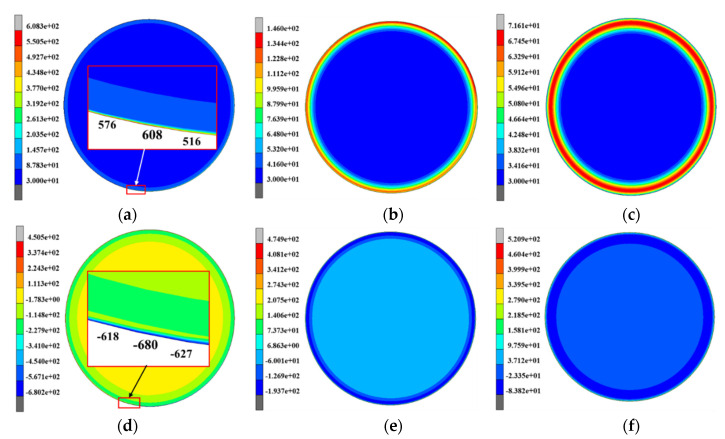
The contours of temperature monitored at the different time points: (**a**) *t*_1_ = 61.06 s (after 30 revolutions of rolling), (**b**) *t*_2_ = 76.03 s (start of idling) and (**c**) *t*_3_ = 105.40 s (end of idling); the contours of thermal stress σ_θ_ monitored at the different time points: (**d**) *t*_1_ = 61.06 s (after 30 revolutions of rolling), (**e**) *t*_2_ = 76.03 s (start of idling) and (**f**) *t*_3_ = 105.40 s (end of idling).

The simulation results both of temperature and thermal stress in Figure 3 indicate that only the outer layer of the roll with a specific thickness experiences considerable temperature and stress changes. This means that the great temperature and thermal stress gradients are contained within a very thin layer which is the so-called thermal skin layer. The thickness of the thermal skin layer is critical to the estimation of thermal behavior of the work roll. In addition to Figure 3, more detailed information on time evolution of the overall temperature and thermal stress fields is given in Figure 4. Figure 4 shows the contours of the temperature and thermal stress monitored at the different time points that are *t*_1_ = 61.06 s (after 30 revolutions of rolling), *t*_2_ = 76.03 s (start of idling) and *t*_3_ = 105.40 s (end of idling), respectively. By comparing the results in Figure 4a–c, these figures confirm that the great thermal gradient remains localized in the thermal skin layer, with an apparent increase of heat penetration in the entire work roll during idling, even though the maximum temperatures and the temperature gradient decrease. Another noteworthy feature is clearly observed: the maximum temperature appears at the roll surface during rolling, but the maximum temperature appears in the subsurface instead of the surface during idling and continuously moves inward. By contrast, it can be found that the maximum thermal stress is always produced at the roll surface, whether during rolling or idling. The distributions of temperature and thermal stress along the radial direction also can be given in another figure.

**Figure 5:**
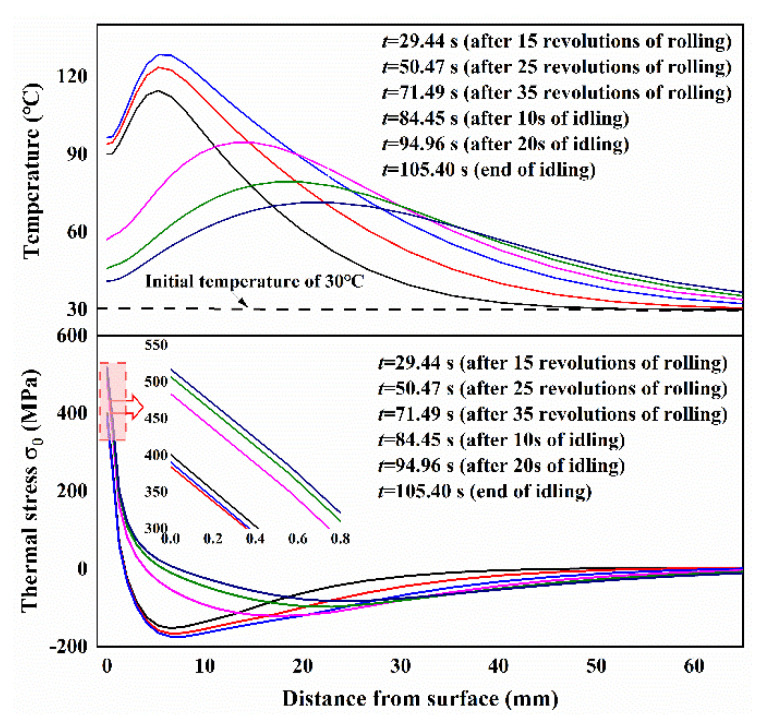
Distributions of temperatures and thermal stresses along the radial direction at different time points.

Figure 5 shows the distributions of temperature and thermal stress along the radial direction at the different instant time points. The results of temperature distribution along the radial direction indicate that the thicknesses of the thermal skin layer are localized at a depth of about 5 mm during rolling and a depth of about 15 mm during idling. Comparing the results of thermal stress during rolling, the tensile stresses at the roll surface gradually increase and the compressive stresses at the subsurface effectively decrease.

### 3.2. Thermo-Mechanical Stress in Work Rolls during Rolling

**Figure 6:**
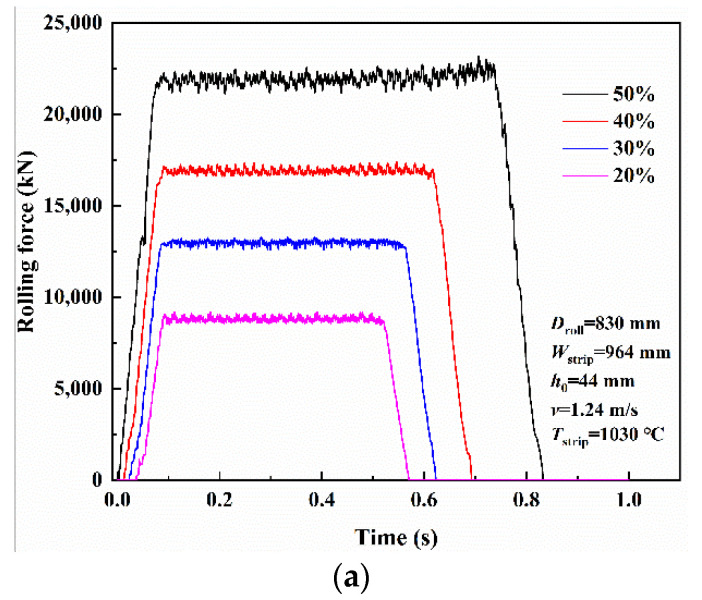

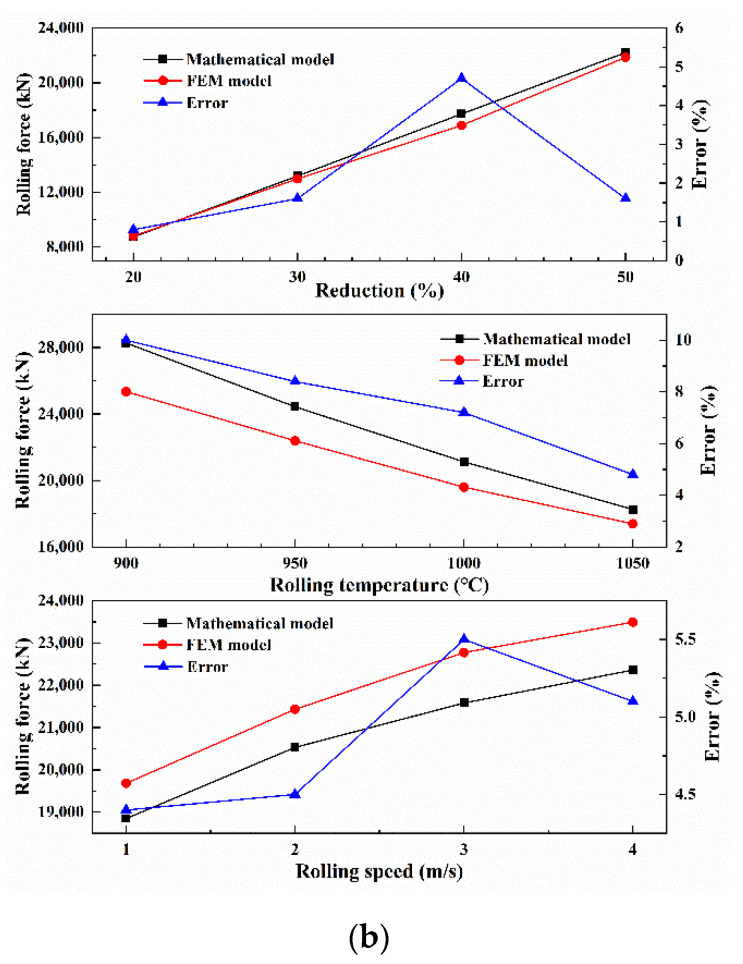
(**a**) Rolling force with time under different reduction, (**b**) comparison of rolling force between the FEM results and mathematical model results.

In the hot rolling process, backup rolls have little effect on the roll force, thus a simplified elastic-plastic FEM model composed of work rolls and strip steel is established in this paper. In order to verify the FEM model, a comparison of the roll force was made between the result predicted by the FEM model and the calculation results of the traditional Sims model [42]. Selected pass is the finishing stand F1—the rolling parameters of the selected pass are shown in Table 1. Note that the strip length is chosen as 500 mm—long enough to obtain a reasonable rolling force record and short enough to minimize analysis time. The variation curves of rolling force with time under different reduction are shown in Figure 6a. In addition, several groups of predicted results considering rolling speed and rolling temperature are also compared with the mathematical model results data as shown in Figure 6b. In contrast to the simulated results and the calculation values, it is known that the two results coincide with each other well, which certifies the feasibility and validity of this FEM model. In practice, roll force is a very complex problem and can be influenced by many factors such as flow stress (influenced by chemical composition, strip temperature, strain rate, strain), friction coefficient (influenced by tribological properties, steel material, workpiece geometry), strip thickness at entry and exit, roll diameter and forward/backward tension and so on. In the current FEM model, some factors have not been considered because this study aims to evaluate the temperature and the coupled thermo-mechanical stress that contributes to the fatigue damage of the work roll under practical industrial service conditions.

**Figure 7:**
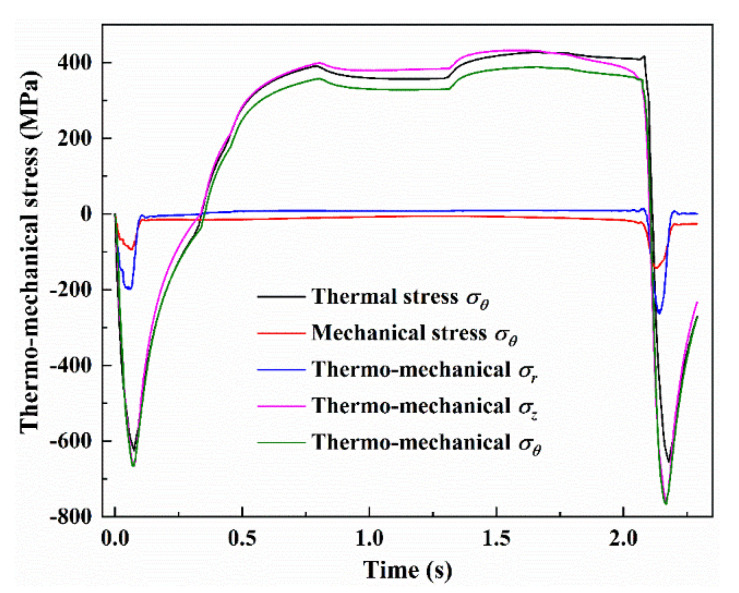
Thermo-mechanical stress components in radial, circumferential and axial directions at the roll surface comparing with thermal stress and pure mechanical stress.

In order to compare with the thermal stress, a simulation of thermo-mechanical stresses in the practical industrial service conditions given in Table 1 was conducted. The roll force obtained from the FEM is 18,630 kN, which shows a good agreement with the experimental result of 19,350 kN. Figure 7 shows simulation results of the thermo-mechanical stress components in radial, circumferential and axial directions at the roll surface. In order to reduce the computational time, only the strip of 4000 mm was performed in this study. In the bite region of the first and second revolution, the maximum circumferential stress related to the thermal load reaches −625 MPa and −655 MPa, whereas the maximum circumferential stress related to the thermo-mechanical load reaches −666 MPa and −766 MPa. The results in Figure 7 indicate that approximately 85% of the magnitude of the compressive circumferential stress at the bite region of the second revolution was caused by thermal loading, and that the only remaining 15% was caused by mechanical loading—6% in the first revolution. This result is consistent with the previous studies which emphasized that 10% of the circumferential stress is caused by rolling pressure [10,19]. As shown in Figure 7, the pure mechanical stress, without considering heat transfer between roll surface and hot strip, is also presented in this paper. By comparison with the calculated values of 41 MPa and 111 MPa of the difference between the thermo-mechanical stress and thermal stress in the bite region of the first and second revolution, the larger, pure mechanical stresses of 102 MPa and 149 MPa are observed. But no matter which stresses are related to mechanical load, the thermal stress is the dominant contribution in the bite region. Note that the magnitude of the maximum circumferential thermo-mechanical stress at the bite region, as well as that at the water spray zone, is very close to that of the axial stress at the same region. However, the radial thermo-mechanical stress is significantly increased compared with the thermal stress.

### 3.3. Redistribution of Heat Treatment Residual Stress in Work Rolls during Hot Rolling

During hot rolling, the roll surface is subjected to the combined effects of thermal stress owing to restriction of the material contraction and expansion, mechanical stress caused by rolling pressure and residual stress produced during the roll manufacturing. Residual stresses are those stresses existing along a cross-section of a component without applied external forces, which are generated during most manufacturing processes involving material deformation, heat treatment, machining or processing operations. Thermal fatigue cracking normally initiates at the roll surface, and can be mitigated by inducing surface compressive residual stresses. The presence of tensile residual stresses in a part or structural element is generally harmful since they can contribute to, and are often the main cause of, fatigue failure and stress corrosion cracking. Assessment of the effect of residual stresses on fatigue crack initiation and propagation then becomes an important aspect of component design and life management. Here, note that two different residual stresses were described: the heat treatment residual stress induced by temperature gradient and phase transformation during heat treatment, and the thermal residual stress caused by great nonuniform temperature distribution that results in plastic deformation over the work roll surface during hot rolling. In order to reveal the stress distribution occurring in actual work rolls, the simulation of the thermal stress should take into account the heat treatment residual stress as the initial stress.

**Figure 8:**
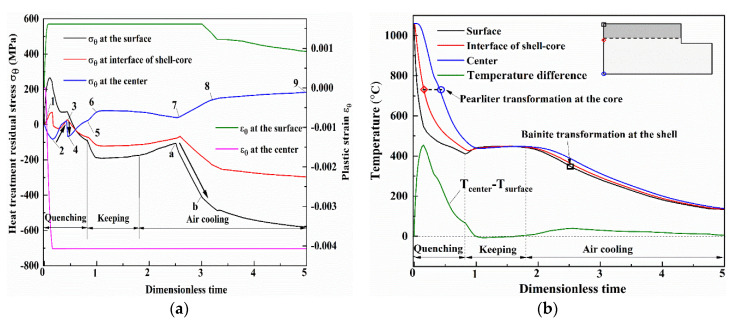
The histories of temperature and heat treatment residual stress at the surface, interface of the shell-core and the center of the work roll during the quenching process: (**a**) temperature and temperature difference between the roll surface and roll center; (**b**) heat treatment residual stress and plastic strain.

In the heat treatment process, the whole roll is firstly heated up to a quenching temperature of 1060 °C. Then, the roll is taken out of the heating furnace and quenched by spray cooling. After this rapid cooling, the roll is maintained for several hours when the surface temperature drops to 450 °C. After the keeping period, the roll is taken out of the furnace and slowly cooled down in air until it reaches room temperature. After the quenching process, the tempering process including heating, keeping at 550 °C and the cooling process, is performed twice to release the residual stress and obtain a stable microstructure. Figure 8 shows the histories of temperature and heat treatment residual stress at the surface, interface of the shell-core and the center of the work roll during the quenching process. The plastic strains at the surface and center in the circumferential direction are also indicated as dashed lines. In the quenching region, the roll surface produces thermal shrinkage with a temperature decrease but it becomes constrained beneath the surface layer with a lower temperature. Consequently, the compressive stress is produced in the roll surface and the contrary tensile stress appears in the center. Both the tensile and the compressive stresses increase when temperature difference between the surface and the center increases (from 1 to 2), resulting in the plastic strains at the surface and center due to the material being soft in high temperature. As the core temperature drops to the temperature of pearlite transformation at point 2, pearlite transformation happens from the interface of the shell-core expanding towards the center (from 2 to 3). Therefore, the compressive stress at the center gradually decreases until tensile stress due to the expansion deformation in the other parts of the roll core is induced by pearlite transformation. Afterwards, the tensile stress rapidly reverses to compressive stress when the pearlite transformation reaches the center (from 3 to 4). After the pearlite transformation, the compressive stress at the center gradually decreases until the tensile stress due to the temperature difference decreases (from 4 to 5). At the beginning of the keeping, the tensile stress further increases at the roll center due to temperature difference decreases caused by the surface heating (from 5 to 6). Then, both the tensile and the compressive stresses gradually decrease due to a slight increase of temperature difference (from 6 to 7). In the subsequent air cooling, bainite transformation occurs at the surface with the volume expansion. As a result, the surface compressive stress increases (from a to b) and exceeds the yield strength of material. To balance the increase in surface compressive stress, the center tensile stress also increases (from 7 to 8). After the bainite phase transformation, the thermal contraction difference increases due to the difference of the thermal expansion coefficients of the two materials, leading to stress increases in the roll surface and center (from 8 to 9).

**Figure 9:**
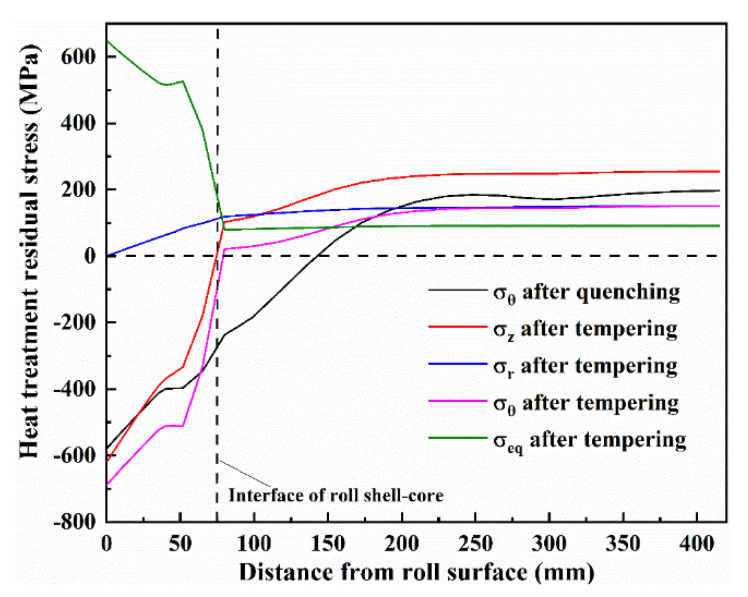
Distribution of the heat treatment residual stress in z, r and θ direction after the tempering process.

In practice, the tempering of the work roll will be conducted after the quenching process to relieve high quenched residual stress. Since the generation mechanism of the heat treatment residual stress is not the focus of this paper, only the final heat treatment residual stress distributions after the tempering process are given. Figure 9 shows the distribution of the heat treatment residual stress in z, r and θ direction after the tempering process. The results indicate that the roll core reserves tensile stress and the roll shell remains under compressive stress after heat treatment. It can be observed that the compressive circumferential stress was larger than the axial stress in the roll shell but tensile circumferential stress was smaller than the axial stress in the roll core. The radial stress was null at the roll surface, which was equal to the circumferential stress in the roll center. By comparing with the heat treatment residual stress after quenching, the heat treatment residual stress in the roll core is effectively reduced by 23% and distributed uniformly after tempering, but the heat treatment residual stress in the roll shell is increased by 19%. These heat treatment residual stresses were inputted into the FEM simulation of hot rolling as the initial stress condition. It should be noted that the coupled residual stress induced by the heat treatment residual stress and the thermal residual stress was simulated, while the mechanical stress was not taken into account because of its small effects and in order to save time.

**Figure 10:**
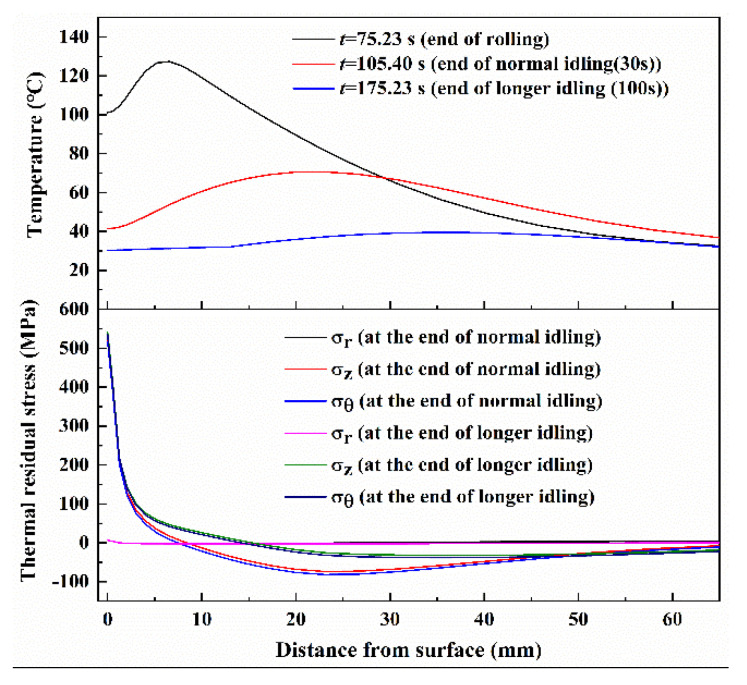
Distribution of the temperature and thermal residual stresses along radial direction at the different instant time points.

As mentioned above, another residual stress, named thermal residual stress, is inevitably produced in the work roll during the hot rolling process. Although the thermal stress has been introduced in Figure 3 and Figure 4, this stress was not equivalent to the final thermal residual stress due to a non-negligible temperature difference existing in the work roll during the hot rolling process, even at the end of idling (see Figure 5). Consequently, a longer idling time (about 100 s) was conducted to obtain a uniform temperature that is close to the ambient temperature in the work roll. In view of the thermal skin where large temperature and thermal stress gradients exist, this study focused on the temperature and the stress in the roll shell. Figure 10 shows the distributions of the temperature and the thermal stress after rolling, normal idling (30 s) and longer idling (100 s), respectively. Comparing with the temperature difference of 96 °C after rolling, the temperature difference in the roll shell gradually decreased to 34 °C after normal idling (30 s) and 10 °C after longer idling (100 s). It can be seen that the tensile residual stresses remained 15 mm beneath the roll surface, and the maximum tensile residual stress reached 535 MPa at the roll surface after longer idling.

**Figure 11:**
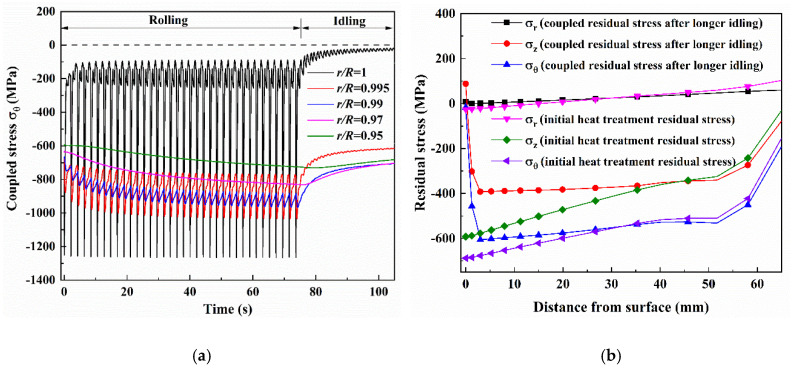
(**a**) Histories of the coupled stress σ_θ_ of thermal stress and heat treatment residual stress with the different depths during the whole hot rolling of one strip and the subsequent idling, (**b**) comparison of initial heat treatment residual stress and coupled residual stress after longer idling.

Figure 11a shows the histories of coupled stress $\sigma_{\theta }$ of thermal stress and heat treatment residual stress with the different depths during the whole hot rolling of one strip and the subsequent idling. The coupled stresses with different depths show a tendency similar to the pure thermal stress in Figure 3, while the whole stresses moved down. It should be noted that no tensile stress was produced with the stress range of −37 MPa~−1212 MPa at the roll surface in the rolling process. During the idling, the compressive coupled stress at the roll surface gradually decreased until it reversed to the tensile stress with the maximum value of 33.8 MPa after normal idling (30 s). This result shows that the heat treatment residual stress can effectively reduce tensile stress during hot rolling. As for the analysis on the elasticity-plasticity behavior and the thermal fatigue life of the work roll being influenced by heat treatment residual stress, this will be left for further research. Figure 11b shows the distributions of the initial heat treatment residual stress and the coupled residual stress after longer idling (100 s). In contrast to the compressive initial heat treatment residual stress, the coupled residual stress significantly decreased to within 3 mm beneath the roll surface. The compressive coupled residual stress decreased and was more uniformly distributed within 3~50 mm beneath the roll surface. In addition, under the cyclic thermal loading, a larger relaxation of axial residual stress was observed in comparison with the circumferential residual stress.

## 4. Conclusions

In this paper, the thermal behavior of a high speed steel work roll during hot rolling and idling is investigated on the basis of the thermo-elastic-plastic finite element method. An FEM model is proposed with an elastic-plastic deformable work roll and a meshed rigid body considering the complex thermal boundary conditions over the roll surface. After that, the thermo-mechanical stress caused by rolling pressure and the redistribution of the initial heat treatment residual stress were investigated. The results of the current study can be summarized as follows.

(1) During the rolling, the maximum temperature and thermal stress in the roll surface increase gradually in the several early revolutions and then reach a stable state. The maximum temperature and thermal stress beneath the roll surface continue to increase, but the amplitudes of temperature variation decrease significantly with the increase of depth. The tensile thermal stress appears in the cooling region of the roll surface without cyclic plastic deformation in this region.

(2) During the idling, the temperature in the roll gradually decreases, but the maximum temperature appears in the subsurface instead of the surface. The compressive thermal stresses turn into tensile thermal stress.

(3) The results of the thermo-mechanical stress indicate that approximately 85% of the magnitude of the compressive circumferential stress at the bite region of the second revolution was caused by thermal loading, and that the remaining 15% was caused by mechanical loading. Thus, the thermal stress makes a dominant contribution in the bite region.

(4) When conducting a longer idling process (100 s) to completely cool the work roll, the tensile residual stresses remain 15 mm beneath the roll surface, and the maximum tensile residual stress reaches 535 MPa at the roll surface.

(5) After heat treatment including quenching and tempering, severe compressive stresses appear at the roll shell and tensile stresses appear at the roll core.

(6) By comparing with the pure thermal stress, coupled stresses, by taking into account heat treatment residual stress, show a similar tendency to the pure thermal stress, while the whole stresses move down and there is no tensile stress during the hot rolling process.

(7) By contrast, compressive initial heat treatment residual stress and the coupled residual stress significantly decrease to within 3 mm beneath the roll surface. The compressive residual stresses gradually decrease to within 3~50 mm beneath the roll surface due to the stress relaxation under the cyclic load.

## Figures and Tables

**Table 1:** Rolling parameters of the finishing stand F1 during hot stir rolling used in the finite element method (FEM) simulation.

Parameters		Value
Velocity of the work roll (m/s)		1.24
Roll diameter (mm)		830
Initial work roll temperature (°C)		30
Entry strip temperature (°C)		1030
Air/water temperature (°C)		30
Entry strip temperature (°C)		1030
Entry strip width (mm)		964
Initial strip thickness (mm)		44
Rolling reduction (%)		43.6
Rolling force (kN)		19,350
Water pressure (MPa)		1.47
Water flow (L/min)		2500
Heat transfer coefficient (W/(m^2^ K))	Bite region	45,000
	Wiper cooling	14,600
	Water cooling	32,600
	Air cooling	5

**Table 2:** Mechanical and thermal properties of the high-speed steel (HSS) work roll at room temperature.

Property	HSS	DCI	Q235
Young’s modulus (GPa)	233	173	210
Poisson’s ratio	0.3	0.3	0.3
Density (kg/m^3^)	7600	7300	7850
Thermal expansion coefficient (/K)	12.6 × 10^−6^	13.0 × 10^−6^	11.9 × 10^−6^
Thermal conductivity (W/(m K))	20.2	23.4	56.9
Specific heat (J/(kg K))	461	460	461
Tensile strength (MPa)	1280	415	235
